# High-Resolution Assembly of the Human Y Chromosome Identifies a Vast Landscape of Inverted Repeats Associated with Structural and Functional Genomic Features

**DOI:** 10.3390/ijms262010180

**Published:** 2025-10-20

**Authors:** Michaela Dobrovolná, Richard P. Bowater, Petr Pečinka, Václav Brázda, Martin Bartas

**Affiliations:** 1 Institute of Biophysics, Czech Academy of Sciences, Královopolská 135, 612 00 Brno, Czech Republic; 2 Faculty of Chemistry, Brno University of Technology, Purkyňova 118, 612 00 Brno, Czech Republic; 3 School of Biological Sciences, University of East Anglia, Norwich Research Park, Norwich NR4 7TJ, UK; r.bowater@uea.ac.uk; 4 Department of Biology and Ecology, Faculty of Science, University of Ostrava, 710 00 Ostrava, Czech Republic

**Keywords:** inverted repeats, human genome, chromosome Y, T2T, bioinformatics, non-B DNA structures

## Abstract

Recent advances in sequencing methods have led to major progress in the gapless assemblies of the human genome. However, until mid-2023, the complete sequence of the Y chromosome remained elusive. While only a small percentage of autosomal chromosomes were without complete sequences in the broadly used reference assembly of the human genome (GRCh38), around 50% of the chromosome Y DNA sequence was unknown. Using a sophisticated computational approach, we analyzed the presence of short inverted repeats in the current human reference genome (GRCh38) and in the Telomere-to-Telomere (T2T) assembly of chromosome Y. This analysis identified the location of the repeats in chromosome Y and highlighted their association with functionally annotated sequences. The comparison revealed notably more inverted repeats in the T2T assembly compared to GRCh38. These are located abundantly around exons and mobile elements, and, unexpectedly, also within gene annotations. The remarkable abundance of short inverted repeats around exons points to their importance in gene regulation, and their presence in regions associated with recombination suggests crucial roles in recombination processes. Interestingly, the most underestimated sequences in the T2T assembly are inverted repeats with a repeat length of 12–14, which are more than 20 times as frequent as those in the human reference genome GRCh38. These findings indicate that the number of short inverted repeats was significantly underestimated in the current human reference genome (GRCh38). These previously unidentified sites are of great bio-medicinal potential, as inverted repeats are precursors for the formation of cruciform DNA functional epitopes.

## 1. Introduction

The Y chromosome, a critical component of the human genome, has long been a subject of scientific interest due to its unique role in determining male sex characteristics and its significant relevance to various aspects of human biology and evolution [1]. The GRCh38 reference assembly has served as a foundational resource in genomics, providing researchers with a comprehensive framework for studying the human genome [2,3]. However, the Y chromosome segment within GRCh38 has presented unique challenges. With approximately 30.8 million bases remaining unidentified, accounting for over 53% of the chromosome, the GRCh38 reference assembly has struggled to capture the complete sequence of the Y chromosome accurately. The gaps in the GRCh38-Y assembly are primarily attributed to the structural complexity of the Y chromosome, specifically the presence of a high proportion of repetitive DNA sequences, including short inverted repeats (IRs), the focus of this study. More complex DNA repeats are found in parts of the chromosome that are annotated as pseudoautosomal regions (PARs), ampliconic regions, and centromeric satellites [4]. Together, these repetitive DNA sequences pose challenges for traditional sequencing methods, leading to gaps and potential assembly errors [5,6]. The incomplete representation of the Y chromosome in GRCh38 has limited our understanding of its function and evolutionary significance.

Recent genomic sequencing breakthroughs [7], exemplified by the Y chromosome telomere-to-telomere (T2T) assembly [4], have significantly enhanced our comprehension of this chromosome and its relevance to the broader human genome. These advancements have notably filled gaps in our knowledge, encompassing previously missing heterochromatic regions, parts of the ampliconic regions, and sequences flanking the endogenous centromere [6]. Unlike the GRCh38-Y assembly, which is known to contain gaps and potential errors, the T2T assembly provides a seamless, gap-free representation of the Y chromosome’s sequence. The completeness of this assembly ensures a more robust foundation for research in male genetics and biology, reducing the likelihood of missing critical genetic information or encountering inaccuracies that could affect scientific conclusions.

**Figure 1:**
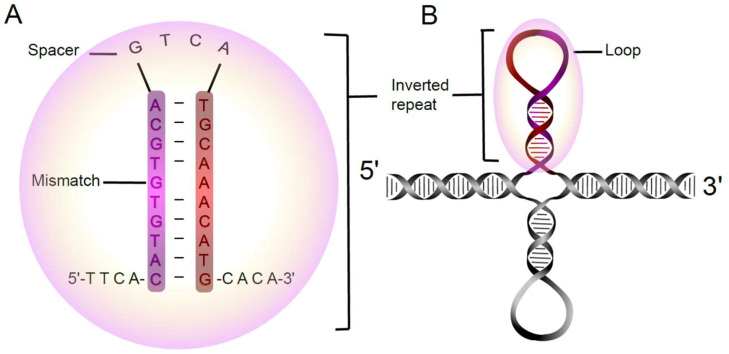
An example of a 10 bp long inverted repeat with one mismatched base pair. The spacer, composed of GTCA, forms a four-nucleotide loop (**A**). The possible formation of a cruciform structure originating from the IRs is shown in panel (**B**).

IRs are important regulatory sequences in genomes with the capacity to form cruciform DNA structures [8]. They are distinctive genetic motifs defined by a sequence of nucleotides followed by a subsequent downstream sequence that mirrors its reverse complement (Figure 1). Thus, IRs involve two sets of nucleotide base sequences in proximity, exhibiting complementary bases when read in the opposite direction. Due to the complementary base-pairing, each IR is able to form a hairpin on its own strand, leading to the formation of a cruciform when these form on both strands. The region between the initial component and its reverse complement is denoted as the gap or spacer of the IR. It is noteworthy that the length of this gap can vary, allowing for flexibility in the overall structure. Remarkably, the gap size may be of any length, including zero, and when it is zero, the entire sequence is regarded as a palindromic sequence [7,9]. The thermodynamic stability of the hairpin and cruciform is related to its number of paired and unpaired bases. Note that mismatches may be tolerated in the hairpin(s), but their presence reduces their thermodynamic stability. Single-molecule experiments and atomistic/coarse-grained simulations indicate that negative supercoiling can induce local unwinding, kinking, and hairpin/cruciform-like transitions in AT-rich sequences at ionic strengths and temperatures comparable to the intracellular environment [10,11]. Several computational approaches have been developed to identify IRs and palindromic DNA motifs across genomes. Earlier tools, such as EMBOSS einverted [12] and IUPACpal [9], provided efficient algorithms for locus-specific searches and motif annotation but are less suited for genome-scale applications. For large-scale comparative analyses, more recent and specialized frameworks—such as Palindrome Analyzer [13]—enable unbiased genome-wide detection of inverted repeats across a broad range of sequence lengths and parameter settings. These developments collectively laid the groundwork for systematic interrogation of inverted repeat architecture at the chromosomal scale. IRs have been detected in all genomes, including viruses [14], bacteria [15], mitochondria [16], and eukaryotic genomes, including the human genome [17,18,19]. Sequences that have the potential to form cruciforms have important regulatory functions, and they are targets of many proteins [20]. On the other hand, the presence of IRs can complicate the assembly process of genome sequences and also impede the study of inversions, duplications, deletions, and rearrangements [21,22]. For example, in the Y chromosome it has been postulated that some repeat regions play important roles in human spermatogenesis, including Azoospermia factors [23].

In summary, the enhanced completeness, accuracy, sequence quality, coverage, and annotation of the T2T Y chromosome assembly made it likely to contain more IRs compared to the GRCh38-Y assembly, as was noted in the study that reported the T2T Y sequence [4]. This improved representation of the Y chromosome’s sequence provides researchers with a valuable resource for exploring the role of IRs across different biological processes [24]. This study delves into the significance of these advancements, improving our understanding of their implications for the field of human health, genetics, and genomics.

## 2. Results

### 2.1. Variation in IR Occurrence and Frequency in Chromosome Y Assemblies

To analyze the presence of IRs in human chromosome Y sequences, we used the Palindrome Analyzer [13] to assess the current reference sequence GRCh38.14p (NCBI ID NC_000024.10) and the T2T Y chromosome assembly (NCBI ID NC_060948.1). A summary of the base sequences identified in the two Y assemblies is presented in Table 1. Note that this data counts only the number of nucleotides in the strand presented in the NCBI database, allowing us to capture information about the imbalance in the nucleotides in individual strands of the Y chromosome. Supplementary Material S01 contains the raw data for all identified IRs in the GRCh38.14p assembly, while Supplementary Material S02 contains the corresponding data for the T2T assembly.

**Figure 2:**
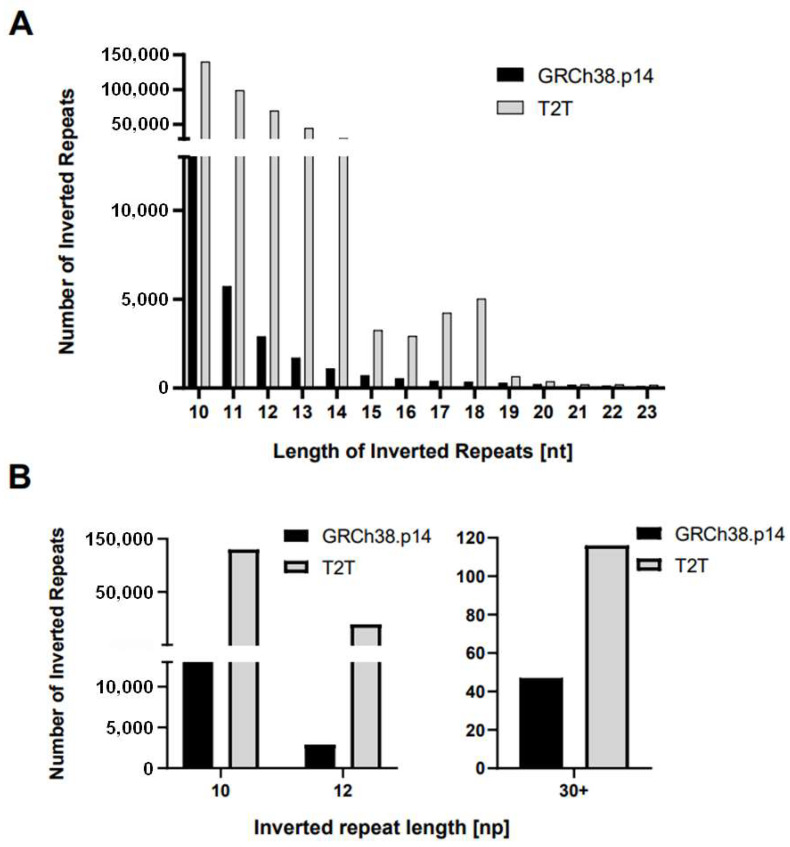
The presence of inverted repeats of different lengths in both human Y chromosome assemblies (**A**) IRs with repeat length 10–23 (**B**) selected lengths only. The size of the bar shows the number of IRs of indicated length in bp of one arm of the repeat in GRCh38.14p (black bar) and T2T (gray bar).

The T2T assembly successfully identified over 30 million nucleotides that were previously unidentified in GRCh38. This updated assembly of the human Y chromosome is more than 5 million nucleotides longer than GRCh38, constituting over 9% of the total length. All nucleotides exhibit an extensive increase in their number in the new assembly compared to GRCh38, although there is some variation in the proportional increase across the different nucleotides (see final column of Table 1). This variation in nucleotide composition, especially in the context of the substantial increase in assembly length, may have implications for the occurrence of repetitive DNA sequences. We then compared the number of IRs in each assembly (Table 2). Compared to the number of newly identified nucleotides in the gapless assembly, there is a much larger proportional increase in inverted repeats for all reported lengths. Interestingly, not all IR lengths have the same proportional increase, with the length of repeats of 12–14 bp having the highest increases (more than 20 times as often in the T2T gapless assembly) (Figure 2A). Note that the number of IRs in the T2T assembly is higher for all lengths analyzed.

In the GRCh38.p14 assembly, the counts of inverted repeats decrease exponentially as the length of the repeats increases. This pattern is expected and aligns with typical genomic characteristics. However, the T2T assembly has an interesting deviation from this trend, specifically, with a notable increase in the counts of IRs at lengths 17 and 18. This observation is depicted in Figure 2A.

IRs of 17 and 18 bp in length for their stem were identified as being enriched in the T2T assembly, so these were selected for further sequence pattern analysis, which was undertaken for repeats with any loop size. This analysis revealed that the majority of these IRs consist of identical or highly similar sequences. Notably, over 88% of the 18 bp IRs are composed of the sequence ‘ATATAATATATATTATAT’, representing 4298 out of the 4865 total IRs with 18 bp repeat. Notably, this IR is composed of 2 shorter perfect IRs of length 9 nt. Transcription factor binding site analysis revealed the presence of several putative protein binding sites in this IR, including Homeobox D8 (HOXD8), Glucocorticoid Receptor Beta (GR-β), POU Class 1 Homeobox 1 (POU1F1s), and Cone-Rod Homeobox (Crx). The two HOXD8 binding sites are particularly interesting because expression of this gene is most enriched in male tissues (Y-linked), according to the Human Protein Atlas [25]. Similarly, the sequence ‘ATGTAATTCTACATATT’ accounts for 3439 out of 4112 sequences, or over 83%, of the 17 bp IRs. Transcription factor binding site analysis revealed the presence of binding sites for GR-β, POU1F1a, and Crx. The exact counts of sequences for all 17 and 18 bp IRs are provided in Supplementary Material S04. To test whether the most abundant short IRs are Y-specific, we quantified exact matches of IRs by length on chromosomes Y, X, and 22 of the T2T reference and normalized the counts by chromosome length (occurrences per 1000 bp). The 17-nt class occurs at 0.068 per kb on chrY versus 0.009 per kb on chr22 and 0.016 per kb on chrX; the 18-nt class occurs at 0.080 per kb on chrY versus 0.007 per kb on chr22 and 0.012 per kb on chrX. Thus, chrY shows a 4–11× higher density of these short AT-rich IRs compared with chrX and chr22. We also report chromosome GC content for context (T2T: chrY = 36%, chrX = 39.5%, chr22 = 46%); because the consensus motifs are highly AT-rich, base composition is expected to contribute to their distribution, but the fold enrichment on chrY is substantially larger than the modest GC differences alone would predict. To evaluate whether this enrichment is statistically significant, we compared the observed IR counts with the expected values, which are proportional to chromosome length. Both the 17 bp and 18 bp IR classes show a strong deviation from the null model of uniform distribution (χ^2^ = 4290.3 and 5910.2, respectively; *p* < 1 × 10^−15^), confirming that these motifs are markedly enriched on the Y chromosome even after accounting for chromosome size. A full table of 1 counts and normalized frequencies is provided in Supplementary Material S04.

In Figure 2B, the distinction in the number of IRs of lengths 10, 12, and 30+ bp is illustrated for both assemblies. Notably, for 10 bp IRs, T2T revealed more than 10 times the number of IRs compared to the reference sequence GRCh38.14p. Furthermore, for 12 bp IRs, the difference was even more pronounced, with T2T exhibiting over 20 times the number of IRs. It is worth highlighting that 116 IRs of 30 bp and longer were identified in the T2T assembly, whereas only 47 of the same lengths were found in the reference sequence GRCh38.14p.

**Figure 3:**
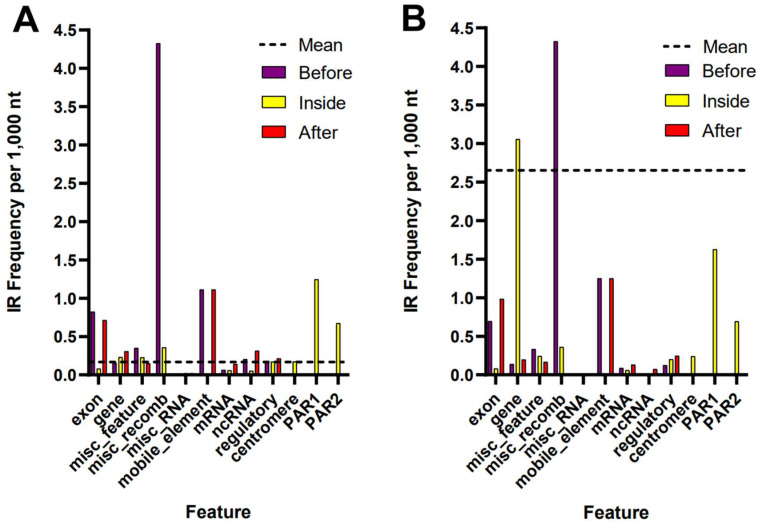
Variations in the frequency of inverted repeats of 12 or more bp across annotated locations in both human Y chromosome assemblies. A black horizontal line represents the frequency across the entire assembly. The frequencies of IRs per kb of sequence are shown within, preceding (100 bp before), and following (100 bp after) the annotated regions for both GRCh38 (**A**) and T2T (**B**) Y chromosome.

**Figure 4:**
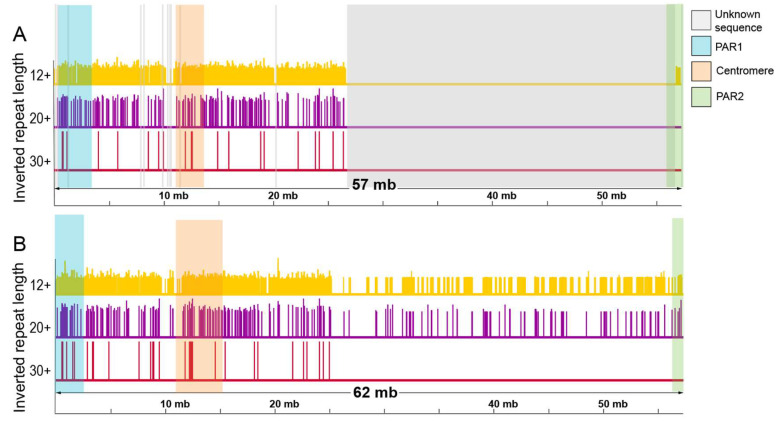
Distribution of inverted repeats across both human Y chromosome assemblies. Comparison of the distribution of all inverted repeats of lengths 12 and longer (yellow lines), 20 and longer (purple lines), and 30 bp and longer (red lines) in GRCh38 (**A**) and the T2T Y chromosome (**B**). The length of the line indicates the length of the IR. The localization of the pseudo-autosomal regions (PAR), unknown sequence, and centromere is shown by colored backgrounds (PARs are blue and green, centromere is light orange, and the unknown sequence region is in gray).

Next, we analyzed the locations of IRs in each assembly sequence. This analysis revealed a depletion in the second half of the GRCh38.p14 assembly, as illustrated in Figure 3A. This underscores that the predominant location of IRs, regardless of their length, was on the short arm of the chromosome. A small number of exceptions to this pattern were observed, involving a few short IRs located at the end of the Y chromosome. Notably, for the T2T assembly, the second half exhibited a significant enrichment of IRs of 12 or more bp, presenting a contrast to the observed pattern in the GRCh38.p14 assembly (Figure 4B). IRs of 20 bp and longer were also found in the second half of the T2T assembly of the Y chromosome, but with a much lower density. Furthermore, IRs of 30 bp and longer were exclusively located in the first half of both assemblies, being absent in their respective second halves. It is also informative to consider how the IRs relate to well-classified complex repeats in the chromosome, such as the two pseudoautosomal regions (PAR1 and PAR2), which are able to pair and recombine during meiosis with the X chromosome [4,26]. PAR1 is located at the terminal region of the short arm, and PAR2 at the tip of the long arm; both PARs and the centromere in both assemblies are shown in Figure 3.

### 2.2. Comparison of IR Occurrence Around Annotated Features of the Genomes

To locate IRs within annotated genomic features, the file containing annotations for known genomic features within the Y chromosome was downloaded from the NCBI database. The existence of IRs within a predefined genomic feature (e.g., gene, mRNA, exon) or within ±100 bp of these genomic features was determined.

Interestingly, a major difference between the assemblies was observed, with an enrichment of IRs within the gene regions for the T2T assembly, with IRs having more than six times the abundance per 1000 bp in the gene region (Figure 3). The higher abundance of IRs in gene regions suggests that, as expected, the T2T assembly provides a more accurate and complete annotation of genes, which opens up possibilities for discovering previously unknown genes, regulatory elements, and genetic interactions. No IRs were identified within and around miscellaneous small RNA (misc_RNA), which encompasses transcripts or RNA products that cannot be classified by other RNA descriptors (e.g., ncRNA, mRNA). While its specific function remains unidentified, misc_RNA has the potential to serve a variety of functions, including possible roles in DNA replication and RNA stability [27,28]. Notably, more IRs were observed in regions lacking annotations. Results for all features for both assemblies are available in Supplementary Material S03.

Even if the frequency of IRs in “genes” is higher compared to other features, it is clear that not all genes contain IRs. Further analyses identified that IRs are present in 104 of 126 characterized genes on chromosome Y (Supplementary Material S06). Contrary to the “gene” annotation data (with an IR frequency of 3.05 per kbp, Figure 3, Supplementary Material S03), the frequency of IRs is almost 40 times lower in the “exon” annotation (0.08 IRs per kbp). However, IRs are more abundant within 100 bp before and after “exon” annotations. Since the NCBI features table did not distinguish the “intron” regions, we downloaded information about some known genes from the ENSEMBL database [29]. The sex-determining region Y (*SRY*) gene is known to be a short, intronless gene that does not contain any introns in its 828 bp sequence. On the other hand, 104 (82.5%) of characterized genes in chromosome Y contain IRs, and 56 genes contain 10 or more IRs. The majority of these IRs are located in introns that are outside of the coding region. For example, the *DAZ1* gene encodes an RNA-binding protein that is important for spermatogenesis [30], and it contains the majority of its IRs in introns (81.8%), with 9.1% of its IRs in exons and 3′-UTR regions. Similarly, 14 IRs were found within the introns of the RPS4Y1 gene (Ribosomal protein S4 Y-linked 1), representing 88% of all IRs in RPS4Y1. Interestingly, the RPS4Y1 gene has been retrotransposed to autosomal regions in several mammals, including opossum, cattle, rat, and mouse [31]. Functional analysis of two gene sets (and “IR-rich” set containing at least 1 IR of length 12+ bp in their stem, and an “IR-poor” set containing 0 IR of length 12+ bp in their stem) revealed several interesting observations, with detailed results together with *p*-values and interaction strengths enclosed in Supplementary Material S07. Firstly, genes containing at least 1 IR of length 12+ were significantly overrepresented in the Gamete generation process (GO:0007276), 3-UTR-mediated mRNA stabilization (GO:0070935), and Alternative mRNA splicing, via spliceosome (GO:0000380). In contrast, the IR-poor group of genes showed strong enrichment for proteins involved in Histone binding (GO:0042393). Secondly, three IR-rich genes (*USP9Y*, *DDX3Y*, *DAZ1*), or their protein products, play a significant role in Sertoli cell-only syndrome (DOID:0050457). Finally, proteins encoded by IR-rich genes show a strong over-representation of the “RNA binding” category, as classified by the UniProt Keyword category (KW-0694). In contrast, the set of IR-poor genes encodes several proteins with Chromatin organization modifier domain (CDY1, CDY1B, CDY2A, CDY2B).

**Figure 5:**
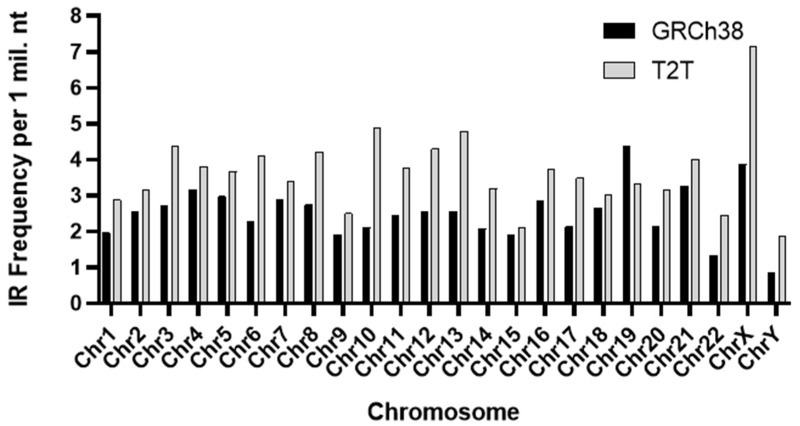
Variations in the frequency per 1 million bp of inverted repeats of length 30 or more for all *H. sapiens* chromosomes for both GRCh38 (black bars) and T2T (gray bars) assemblies. Frequencies across the two assemblies were found to be significantly different from one another (*p* < 0.0001) as determined via a non-parametric Wilcoxon Signed-Rank Test for Paired Samples.

A significant difference in the frequency of IRs, each spanning 30 bp or longer, was observed across chromosomes in the two assemblies (*p* < 0.0001), as shown in Figure 5. Notably, a higher prevalence of IRs was noted in the T2T assembly. Interestingly, at a proportional level, chromosome Y was found to be the least enriched with IRs in both assemblies.

## 3. Discussion

**Figure 6:**
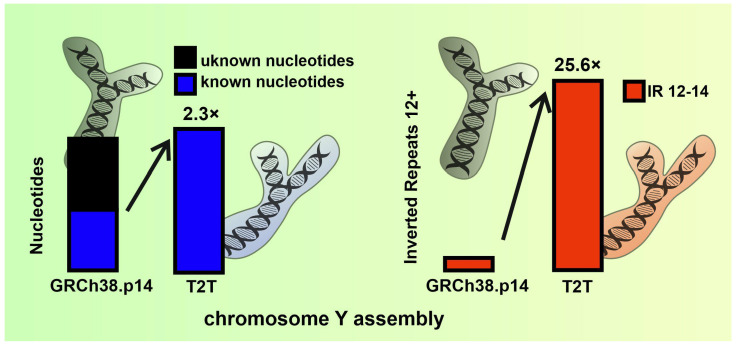
Comparison of human chromosome Y assemblies revealed that the telomere-to-telomere (T2T) assembly contains more than twice as many known bp compared to the current reference assembly GRCh38 (left part, blue). These newly sequenced parts are abundant with 12–14 bp inverted repeats, with the Palindrome Analyzer showing they contain more than 20 times as many inverted repeats compared to the reference assembly GRCh38 (right part, red).

The use of advanced sequencing technologies recently allowed the completion of the entire human genome sequence, nearly without any unidentified parts [2]. The biggest remaining challenge was the assembly of chromosome Y, where more than half of the sequences were unknown in the previous reference assembly. However, even this challenging aspect of the genome, with many repeat and duplication regions [32,33], was finally solved in 2023 [4]. The male-specific region of the human Y chromosome contains many amplicons whose sequence similarity is maintained by interlocus gene conversion [34,35]. These changes are, on the one hand, important in evolution, but on the other hand, they are associated with various sex-associated disorders, including male infertility, Turner syndrome, and sex reversal [36]. Therefore, detailed knowledge of repetitive sequences in chromosome Y is very important for understanding the evolutionary development of these disorders. Recently, the strong abundance of G-quadruplex-forming sequences in chromosome Y has been shown [37]. Notably, the q-arm of the human chromosome Y contains an enormous amount of complex tandem repeats. For example, nested tandem repeats of the DYZ2 satellites are extremely AT-rich regions (over 85% AT) that cause an imbalance in the GC content of chromosome Y in comparison to other human chromosomes [4]. Comparative analysis of IR patterns between the GRCh38.14p and the T2T Y chromosome assemblies revealed notable differences in terms of their occurrence, frequency, and distribution. These findings shed light on intricate structural variations within the Y chromosome, highlighting potential implications for functional genomics. Interestingly, only the relatively short inverted repeats (lengths 12–14 bp) were extremely abundant compared to the previous assembly (Figure 6). The substantial increase in the abundance of IRs per 1000 bp in gene regions of the T2T Y assembly raises intriguing questions about the role of IRs in transcription and their regulation.

The analysis of IR locations in Y chromosomes revealed notable shifts in distribution, with a depletion of IRs in the second half of the GRCh38.p14 assembly and an enrichment of short and intermediate-length IRs in the corresponding region of the T2T assembly. The short (17–18 nt), AT-rich IRs are particularly over-represented on chrY—occurring at several-fold higher density than on chrX or chr22—and dominated by two highly repetitive motifs (atataatatatattatat and atgtaattctacatatt), consistent with a Y-specific expansion or clustering of AT-rich satellites [4]. Although differences in GC content partly explain this bias, the magnitude of enrichment indicates that localized repeat accumulation, rather than base composition alone, drives their Y-linked abundance. One plausible explanation for the elevated counts of these short IRs in the T2T assembly is that they arise from regions of structural variation or genomic instability that were previously unresolved or collapsed in earlier references. Such AT-rich segments are inherently prone to helix destabilization and local unwinding, favoring the extrusion of secondary structures such as hairpins or cruciforms. These features may, in turn, contribute to the high structural plasticity and rearrangement potential characteristic of the Y chromosome. Alternatively, the enrichment may reflect Y-specific repetitive elements or sequence motifs that have expanded or been selectively maintained in the fully resolved T2T sequence. While our current data do not permit definitive conclusions regarding their functional significance, the inherent instability and extreme AT content of these regions suggest possible roles in replication dynamics, chromatin organization, or structural maintenance of the Y chromosome. Highlighting these AT-rich inverted repeats and their potential contribution to Y-chromosome evolution and architecture provides an important direction for future investigation. This observed structural variation suggests nuanced differences in Y chromosome organization between the two assemblies. Previous studies of non-B DNA structures in the gapless assemblies of chromosome 8 and chromosome X revealed a similar trend, namely that sequences with the potential to form non-B DNA structures, such as G-quadruplexes and cruciforms, were underestimated in the previous assemblies [38,39]. However, the presence of IRs in human chromosome Y is very specific, and in this study, we found noteworthy increases in the abundance of IRs in the fully sequenced version of this chromosome. These findings highlight the need for additional laboratory- and bioinformatics-focused studies to gain a deeper understanding of the distribution and function of repetitive DNA sequences across all chromosomes in the human genome.

An inseparable part of the story is the interaction between IRs and transcription factors or proteins in general. As IRs can often form cruciform DNA structures, they can attract a wide range of structurally specific proteins [20]. In addition, the IRs may contain known sequential transcription factor binding motifs, as mentioned in the Results section above. Therefore, IRs across the complete human genome could function as sequestering sites for particular transcription factors and further affect mechanical forces driving genome condensation and rearrangements [40].

It is notable that IRs are often considered to be sites of genomic instability [24,41] and such sites can be polymorphic between various individuals and species. For instance, the overlap between mirror and inverted repeats on the Y chromosome is less pronounced in non-human apes than in humans, which likely reflects species-specific variations in the satellite content of the Y chromosome [42]. This variation is also evident in human Y chromosome phylogenies, where unexplained branch length variation is observed, particularly in lineages that are highly divergent from the human reference Y chromosome. Such variation highlights the importance of refining our understanding of human evolutionary history, which in turn facilitates more accurate reconstructions of demographic events [43].

Therefore, as novel versions of gapless assemblies from human individuals across (sub) populations are finalized, interesting and novel correlations will probably be revealed, allowing connections of particular IR expansion or deletion to physiological conditions/disease. In other words, each of the newly identified IRs on chromosome Y could represent a novel candidate regulatory site.

## 4. Materials and Methods

### 4.1. Genomes

The complete chromosome Y sequences were retrieved from the National Center for Biotechnology Information (NCBI) database. Specifically, this study focused on the current reference sequence GRCh38.14p (NCBI ID NC_000024.10) [33] and the recently released telomere-to-telomere Y chromosome assembly (NCBI ID NC_060948.1) [4].

### 4.2. Analyses of Short Inverted Repeats

Palindrome analyzer [13] was used for the determination of the presence of IRs with the following parameters: size 10–30 (length in bp of one arm of the repeat), spacer 0–10, and a maximum of 1 mismatch in the stem. All sequences were analyzed in the 5′-3′ direction. By setting this relatively high threshold, we aim to filter out shorter inverted repeats that might appear frequently but lack significance. This approach helps us focus on more meaningful and dependable data points, enhancing the quality and relevance of our findings. The complete results of these analyses can be found in Supplementary Material S01 (for GRCh38.14p) and in Supplementary Material S02 (for telomere-to-telomere Y chromosome assembly). For both assembly results, BedGraph files are enclosed in Supplementary Material S08. Separate tracks are provided for different IR length classes and can be visualized directly in genome browsers.

### 4.3. Analyses of Genomic Features Overlap with Inverted Repeats

Annotations for genomic features within the genomes of the Y chromosome were downloaded from the NCBI database. Integrative Genomics Viewer (IGV) [44] was employed to depict the distribution of IRs across the chromosomes, while other charts were generated using GraphPad Prism (version 10). The localization of the pseudo-autosomal regions (PAR) and centromere was obtained from the NCBI genome browser. Detailed annotation of genomic features overlap is enclosed in Supplementary Material S03.

### 4.4. Sequence Identity Analyses and Transcription Factor-Binding Sites Prediction

IRs of 17 and 18 bp in length (with all kinds of loops) identified in the T2T assembly were selected for further sequence pattern analysis. This analysis was performed using a custom Python script (Python version 3.8.6), and the results are available in Supplementary Material S04. Transcription factor binding sites were predicted within a dissimilarity margin of less than or equal to 15% using PROMO [45,46]. A detailed graphical representation of the results is provided in Supplementary Material S05.

### 4.5. Statistical Evaluation

Normality of the data was determined via a Shapiro–Wilk test. To determine significance, the Wilcoxon Signed Rank Test for Paired Samples was used. All graphs were generated using GraphPad Prism. Functional analysis and statistical evaluation of genes that are rich or poor in IRs was performed using the STRING tool (v. 12.0) with default parameters [47].

## Figures and Tables

**Table 1:** Basic sequence characteristics in assemblies of the human chromosome Y DNA sequences, GRCh38.14p (NCBI ID NC_000024.10) and T2T Y chromosome assembly (NCBI ID NC_060948.1). The details presented here show only the number of nucleotides in the strand presented in NCBI. Additionally, the table presents differences between the two sequences, Δ (T2T − GRCh38.p14) and Δ% ((T2T − GRCh38.p14)/GRCh38.p14). * Since many nucleotides were unknown (N) in assembly GRCh38, the GC content is based on known bases, and the number of GC is not divided by the chromosome length but by the number of known bases.

	GRCh38.p14	T2T	Δ	Δ%
Length	57,227,415	62,460,029	5,232,614	9.1
A	7,886,192	21,954,563	14,068,371	178.4
T	7,956,168	17,929,049	9,972,881	125.4
G	5,286,894	13,373,414	8,086,520	152.9
C	5,285,789	9,203,003	3,917,214	74.1
N	30,812,372	0	−3.1 × 10^7^	−100
GC	10,572,683	22,576,417	12,003,734	113.5
GC [%]	40.03 *	36.15	−3.88	−9.7

**Table 2:** Identification of inverted repeats in assemblies of the human chromosome Y DNA sequence. Inverted repeats found in Y assembly sequences (GRCh38.14p (NCBI ID NC_000024.10) and T2T Y chromosome assembly (NCBI ID NC_060948.1)) are categorized by their length—counts of inverted repeats falling into length groups (A), counts of inverted repeats with specific lengths (B), and IRs frequency per 1 kbp (C). Additionally, the table presents differences between the two sequences, Δ (T2T − GRCh38.p14) and Δ% ((T2T − GRCh38.p14)/GRCh38.p14).

A				
IR length	GRCh38.p14	T2T	Δ	Δ%
all	28,003	399,394	371,391	1426.3
12+	8962	160,726	151,764	1793.4
20+	982	1423	441	144.9
B				
IR length	GRCh38.p14	T2T	Δ	Δ%
10	13,313	139,977	126,664	1051.4
11	5728	98,691	92,963	1723
12	2898	69,430	66,532	2395.8
13	1700	44,452	42,752	2614.8
14	1096	29,347	28,251	2677.6
15	713	3247	2534	455.4
16	542	2924	2382	539.5
17	396	4227	3831	1067.4
18	341	5023	4682	1473
19	294	653	359	222.1
20	213	373	160	175.1
21	176	195	19	110.8
22	136	194	58	142.6
23	100	160	60	160
24	104	126	22	121.2
25	66	80	14	121.2
26	48	56	8	116.7
27	41	43	2	104.9
28	30	45	15	150
29	21	35	14	166.7
30+	47	116	69	246.8
C				
IR length	GRCh38.p14	T2T	Δ	Δ%
all	0.489	6.394	5.915	1306.8
12+	0.157	2.573	2.417	1643.2
20+	0.017	0.023	0.006	132.8

## Data Availability

The data presented in this study are available in the Supplementary Materials.

## Supplementary Materials

The following supporting information can be downloaded at: https://www.mdpi.com/article/10.3390/ijms262010180/s1.

## Abbreviations

The following abbreviations are used in this manuscript:

bp	Base Pair
Chr	Chromosome
Crx	Cone-Rod Homeobox
DAZ1	Deleted in Azoospermia 1
DOID	Disease Ontology Identifier
DNA	Deoxyribonucleic acid
ENCODE	Encyclopedia of DNA Elements
ENSEMBL	European Bioinformatics Institute Genome Database
GC	Guanine–Cytosine (content)
GR-β	Glucocorticoid Receptor Beta
GRCh38	Genome Reference Consortium Human Build 38
HOXD8	Homeobox D8
IGV	Integrative Genomics Viewer
IR(s)	Inverted Repeat(s)
kbp	Kilobase Pair
KW	UniProt Keyword
misc_RNA	Miscellaneous Small RNA
mRNA	Messenger RNA
ncRNA	Non-coding RNA
NCBI	National Center for Biotechnology Information
nt	Nucleotide
PAR(s)	Pseudoautosomal Region(s)
POU1F1a/POU1F1s	POU Class 1 Homeobox 1 (isoforms a/s)
p-value	Probability Value
PROMO	Transcription Factor Binding Site Prediction Tool
RNA	Ribonucleic Acid
RPS4Y1	Ribosomal Protein S4, Y-linked 1
SRY	Sex-determining Region Y
STRING	Search Tool for the Retrieval of Interacting Genes/Proteins
T2T	Telomere-to-Telomere
UTR	Untranslated Region
USP9Y	Ubiquitin-Specific Peptidase 9, Y-linked
