# Supplementary material for: High-Resolution Assembly of the Human Y Chromosome Identifies a Vast Landscape of Inverted Repeats Associated with Structural and Functional Genomic Features
Source: Int J Mol Sci. 2025 Oct 20;26(20):10180. doi: 10.3390/ijms262010180 (PMC12563786; doi:10.3390/ijms262010180)

Supplementary Material 05 - Graphical representation of the complete PROMO transcription binding sites prediction for the two most enriched IRs of length 17 and 18 bp. (A) Transcription factor binding sites for the most enriched 18 bp IR. (B) Transcription factor binding sites for the most enriched 17 bp IR.

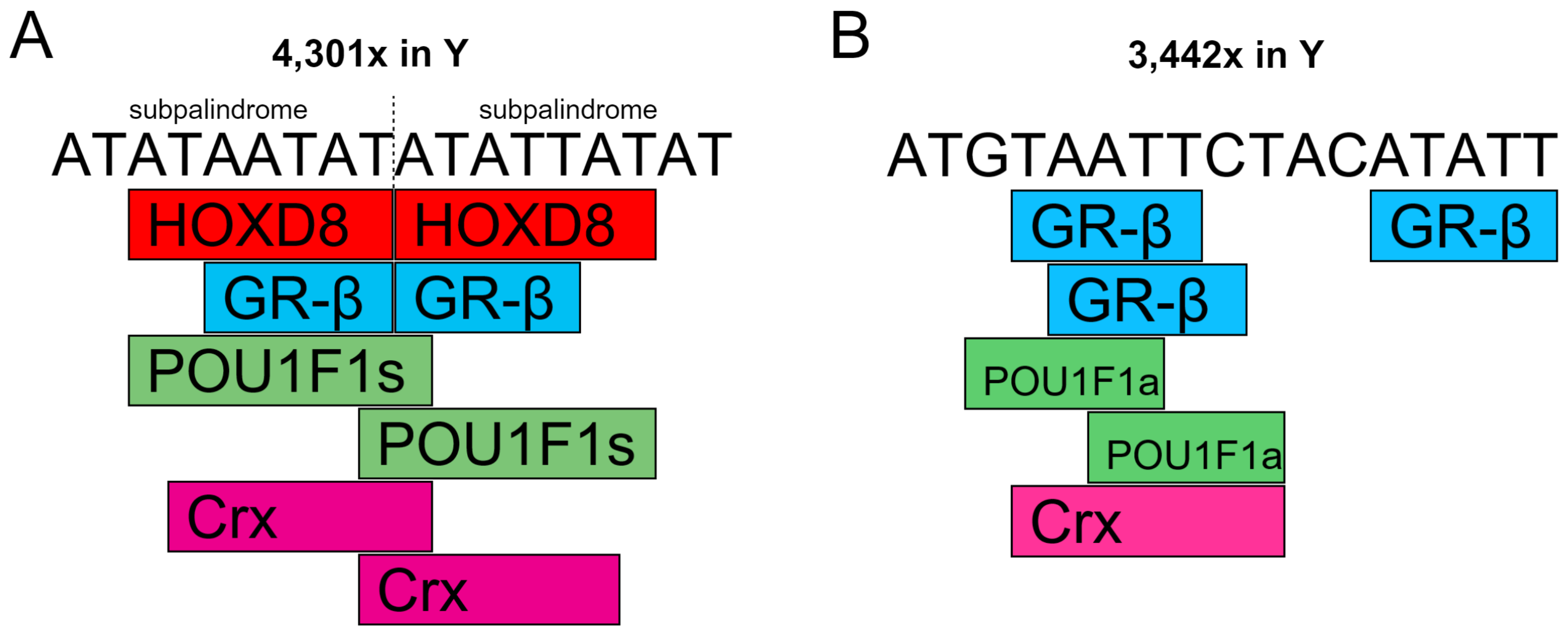

Supplement: Supplementary file 1 [file ijms-26-10180-s001.zip › ijms-3884539-supplementary/Supplementary_Material_05.pdf]
